# Theta-Burst Stimulation for Auditory-Verbal Hallucination in Very-Late-Onset Schizophrenia-Like Psychosis—A Functional Magnetic Resonance Imaging Case Study

**DOI:** 10.3389/fpsyt.2020.00294

**Published:** 2020-04-20

**Authors:** Rebecca Zöllner, Anne-Friederike Hübener, Udo Dannlowski, Tilo Kircher, Jens Sommer, Maxim Zavorotnyy

**Affiliations:** ^1^ Department of Psychiatry and Psychotherapy, University of Marburg, Marburg, Germany; ^2^ Marburg Center for Mind, Brain and Behavior – MCMBB, University of Marburg, Marburg, Germany; ^3^ Health Protection Authority, Frankfurt am Main, Germany; ^4^ Department of Social Psychiatry, University of Applied Science Niederrhein, Krefeld, Germany; ^5^ Department of Psychiatry and Psychotherapy, University of Muenster, Muenster, Germany; ^6^ Core-Unit Brainimaging, Faculty of Medicine, University of Marburg, Marburg, Germany; ^7^ Department of Psychiatry and Psychotherapy, Psychiatric Services Aargau, Academic Hospital of the University of Zurich, Brugg, Switzerland

**Keywords:** brain stimulation, elderly, very-late-onset schizophrenia-like psychosis, auditory verbal hallucination, theta-burst stimulation, functional MRI, auditory processing

## Abstract

**Background:**

Treating very-late-onset (>60 years) schizophrenia-like psychosis (VLOSLP) is challenging. Age-related factors in elderly individuals (e.g., metabolism, medication side effects, drug-interaction, somatic morbidity) may adversely affect treatment. Novel therapeutic approaches are needed to ensure the favorable therapeutic outcome in geriatric patients. Previously, theta-burst stimulation (TBS), a novel form of repetitive transcranial magnetic stimulation, was reported being beneficial in the treatment for auditory-verbal hallucination (AVH) in young and middle-aged schizophrenia (SZ) patients.

**Case Presentation:**

Here we present a case of a male patient aged 73. His first psychotic episode manifested with paranoid delusions, auditory-verbal and tactile hallucinations at the age of 66, and first remitted following a second-generation antipsychotics (SGA). Years later, after a relapse the AVH did not respond to previously effective olanzapine, whereas its augmentation with an inhibitory TBS over the left temporal lobe led to a stable remission. During his second relapse, TBS was again capable of facilitating therapeutic action of SGA in the same patient. Extending to our clinical observation, a series of functional MRI scans employing a tonal activation paradigm depicted altered auditory processing during AVH as well as brain activation change during remission.

**Conclusions:**

The current case might indicate to favorable effects of combining conventional medicament therapy and non-invasive brain stimulation techniques for elderly patients. Also, we speculate that despite obviously distinct etiologies, the present functional imaging and clinical observation may also demonstrate a possible common pathophysiological pathway underlying AVH in VLOSLP and SZ.

## Background

Very-late-onset schizophrenia-like psychosis (VLOSLP) was first described as paraphrenia senilis (1). With its 12-month prevalence of 0.6 %, VLOSLP represents a relatively common mental illness in individuals aged above 60 years (2). Currently, limited data is available on the etiology of VLOSLP, suggesting neurodegenerative and neurovascular origins, as well as the interaction between an accelerated aging (3) process and premorbid genetic and socio-cultural vulnerability (4–6). The core symptoms of VLOSLP are auditory-verbal hallucinations (AVH) and paranoid delusions, which first manifest in the elderly (4, 7, 8). For schizophrenia (SZ) manifesting first in younger age, altered brain connectivity resulting from an abnormal neural maturation has been assumed (9). In particular, altered white matter tracts, such as the uncinate fasciculus (connecting parts of the limbic system with the temporal and orbitofrontal cortex), are involved in the formation of AVH in SZ (10–12). Impaired connectivity of the auditory-related neural circuitry leads to altered self-monitoring, and misinterpretation of own thoughts as external voices (12, 13). Considering their similar psychopathology and responsiveness to the antipsychotic treatment (4, 14), we speculate that AVH in both mental conditions might be caused by disturbances in a common pathophysiological pathway, despite obviously distinct etiological origins of SZ and VLOSLP.

Based on the principle of electromagnetic induction, repetitive transcranial magnetic stimulation (rTMS) as well as its form theta-burst stimulation (TBS) modulate the cortical excitability temporarily (15) and also have long-lasting neural effects (16) allowing to be used as a powerful investigation tool for the functional mapping of the brain. Both rTMS and TBS are well tolerated and have been proposed as a possible novel therapeutic strategy in geriatric psychiatry (17). Its antidepressant action has been described in elderly patients suffering from major depression (18), or for depressive symptoms accompanying Parkinson's disease (19), and stroke (20). Recently, rTMS /TBS has been discussed to become a treatment option for managing positive and negative symptoms in schizophrenic patients (21). The promising clinical application of rTMS /TBS on AVH in early-onset SZ has recently been reported (22–25). Favorable effects for treating AVH with rTMS /TBS in middle-aged SZ and late-onset SZ were also indicated by two previous case reports (26, 27).

Since rTMS /TBS seems to have beneficial effects for managing AVH in schizophrenic patients with early or middle-aged onsets, the question arises whether this non-invasive brain stimulation technique may also be useful for managing AVH in VLOSLP. Thus, we report a case of successful application of add-on inhibitory TBS over the left temporal lobe in an elderly patent with VLOSLP (aged 73). The remission of AVH was achieved twice following a TBS augmented therapy with second-generation antipsychotics (SGA). To explore possible neural similarities with early-onset SZ, we performed an additionally series of functional magnetic resonance imaging (fMRI) scans using an auditory stimulation paradigm contrasting neural activity patterns during the patient's experience and absence of AVH.

## Case Presentation

Mr. WF was born in 1940 in the former Soviet Union in a Russian-German family and immigrated to Germany in 1996. He is right-handed, a German and Russian native speaker with an educational level of 10 years. Before his first psychotic episode, Mr. WF had no history of mental disorders and exhibited a generally sufficient functioning level. Before immigration, he worked as a truck driver. In Germany, he was employed as a caretaker in a school until he was retired at the age of 65. He was married twice and had two adult sons. After his last divorce, he lived in a house he owned, together with one of his two sons. His first psychotic episode occurred in 2006 with paranoid delusions, auditory-verbal and tactile hallucinations. He attacked his daughter-in-law because he misjudged her as a witch and was therefore admitted for psychiatric in-patient treatment. The psychotic symptoms were first treated with risperidone, which was not tolerated by the patient due to extrapyramidal motor side effects. Finally, he remitted following a therapy with olanzapine (15 mg/d). The remission lasted until 2014, although the antipsychotic medication was discontinued shortly after discharge and no out-patient treatment took place.

In April 2014, Mr. WF (now aged 73) was admitted to the Department of Neurology of Marburg University because of dizziness. After the physical causes excluded, and because Mr. WF complained of imperative and commentating voices of two witches, who—as he thought—were responsible for the dizziness, a second psychiatric in-patient admission took place. At the admission to the Department of Psychiatry and Psychotherapy of Marburg University, he experienced AVH, paranoid delusions, and perceived the control by witchcraft. In his physical examination, we saw a right-dominated tremor of the hands. Comprehensive technical examinations (including blood tests, structural MRI, cerebrospinal fluid diagnostics, FP-CIT, and 18F-FDG-brain-PET) depicted a moderate subcortical arteriosclerotic encephalopathy, which might be the cause of the vascular parkinsonism. Considering moderately elevated tau protein levels in the CSF, a mesial temporal reduced 18F-FDG utilization [NIA-AA Biomarker profile A-T+N+ (28)], as well as the impaired short-term verbal and figurative memory, attention, and psychomotor speed, without incapacity for independence in everyday activities, we suggested a minor non-Alzheimer neurocognitive disorder, besides the VLOSLP. For the neurocognitive assessment, the standardized Consortium to Establish a Registry for Alzheimer's Disease (CERAD)-test battery was applied (29); scores are listed in the **Supplemental Material**, **Tables S1** and **S2**. A monotherapy with olanzapine (up to 20 mg/d) led to a remission of perception of control by witchcraft and dizziness. However, the AVH remitted only partially. Since no complete remission could also be achieved during the 12 weeks of further treatment, Mr. WF was re-admitted to our in-patient unit for an add-on inhibitory TBS trial (July 2014). After the first five sessions, the patient reported a significant reduction of the AVH; after ten sessions, a stable remission was achieved.

After the discharge, Mr. WF resigned the out-patient psychiatric treatment and chose to discontinue his antipsychotic medication in November 2014, mostly due to the absence of any subjective complaints. In January 2015, he was again admitted to a neurologic hospital because of dizziness. Due to low body rigidity and tremor, a treatment trial with levodopa was initiated by colleagues. This medication was well tolerated but did not lead to any significant clinical effects. In June 2015, Mr. WF reported a sudden re-occurrence of AVH and paranoid delusions (evaluated by a face-to-face interview using simple YES/NO questioning, see **Supplementary Material Box S1**); thus, a psychiatric re-admission was again required. Since no remission could be achieved after dopamine-agonistic medication and an 123I-N-ω-fluoropropyl-2β-carbomethoxy-3β-(4-iodophenyl)nortropane) (FIT-CT)/single photon emission computed tomography (SPECT) revealed the non-altered density of dopamine transporter, levodopa was tapered out. Next, TBS monotherapy was carried out in July 2015, however, without significant improvement (evaluated by targeted questioning).

Later, we prescribed clozapine (up to 150 mg/d, July 2015), however, failing to reduce the AVH sufficiently (evaluated by targeted questioning), and leading to a psychomotor retardation and further cognitive decline. Thus, clozapine was then tapered out. In August 2015, olanzapine was prescribed again due to prior positive experience with it during the first psychotic episode. The olanzapine monotherapy (4 weeks, 15 mg /d) led again to a merely partial response (evaluated by targeted questioning). Considering the positive experience during the previous in-patient treatment, the TBS augmentation (September 2015) was then repeated and led to a complete remission of AVH (evaluated by targeted questioning) as during the last episode. Later, the remission remained stable in combination with the maintenance medication olanzapine (5 mg/d). An overview of the full treatment course can be seen in the **Supplementary Material**. All essential milestones related to the diagnoses and interventions are presented there as a timeline.

### Theta-Burst Stimulation

**Figure 1:**
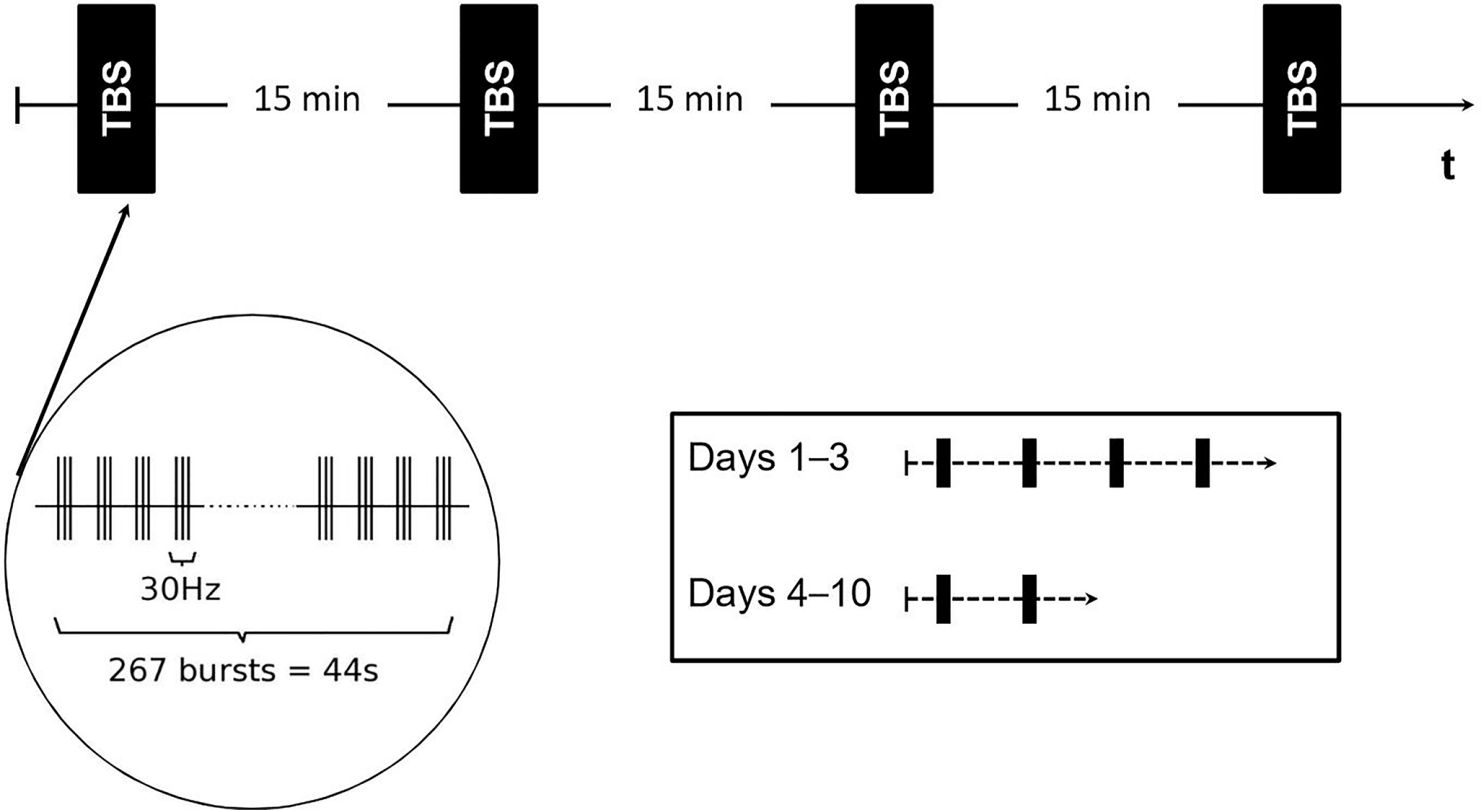
Schematic depiction of the theta-burst stimulation (TBS) protocol on days 1–10. The black rectangle represents one stimulus block. Each block was separated by an inter-stimulus interval of 15 min. On days 1–3, a series of four stimulation blocks were applied while on days 4–10, two stimulation blocks were administered consecutively.

The patient was treated using the MagVenture’s apparatus MagPro X100 with the figure-eight-shaped coil MCF-B65. During all three courses, Mr. WF was stimulated over 10 days (2 x 5 working days) using an inhibitory TBS protocol, as described previously (30, 31). The rationale for the TBS protocol was a considerable reduction of the time of stimulation, which might lead to better acceptance, in particular, for less resilient individuals. A shorter application time and previously reported long lasting effects at the reduction of auditory verbal hallucination (AVH) symptoms (30, 31) has led the decision for the chosen TBS protocol. In brief, a single TBS train lasted 44 s (a continuous train of 801 pulses) consisting of 267 bursts (each burst comprised three pulses at 30 Hz with an inter-stimulus interval of 100 ms). The TBS protocol on days 1–3 included two double trains (4 x 801 pulses; resulting in 3,204 pulses), while on days 4–10, one double train (2 x 801 pulses resulting in 1,602 pulses) was applied. An inter-stimulus-interval of 15 min separated each train. We apply the stimulation over TP3 [defined as half the distance between T3 and P3 according to the international 10–20 EEG system; (32)] and at 90 % of the motor threshold (MT). The TP3 area represents a conventionally targeted region for treating AVH in SZ patients (30, 31, 33). MT was determined for the left abductor pollicis brevis muscle and defined as the minimal stimulus intensity that produces a motor evoked potential in at least 50 % of 10 transcranial magnetic stimuli as described previously (34). **Figure 1** gives an overview of the TBS protocol used here.

### MRI Data Acquisition and Data Analysis

**Figure 2:**
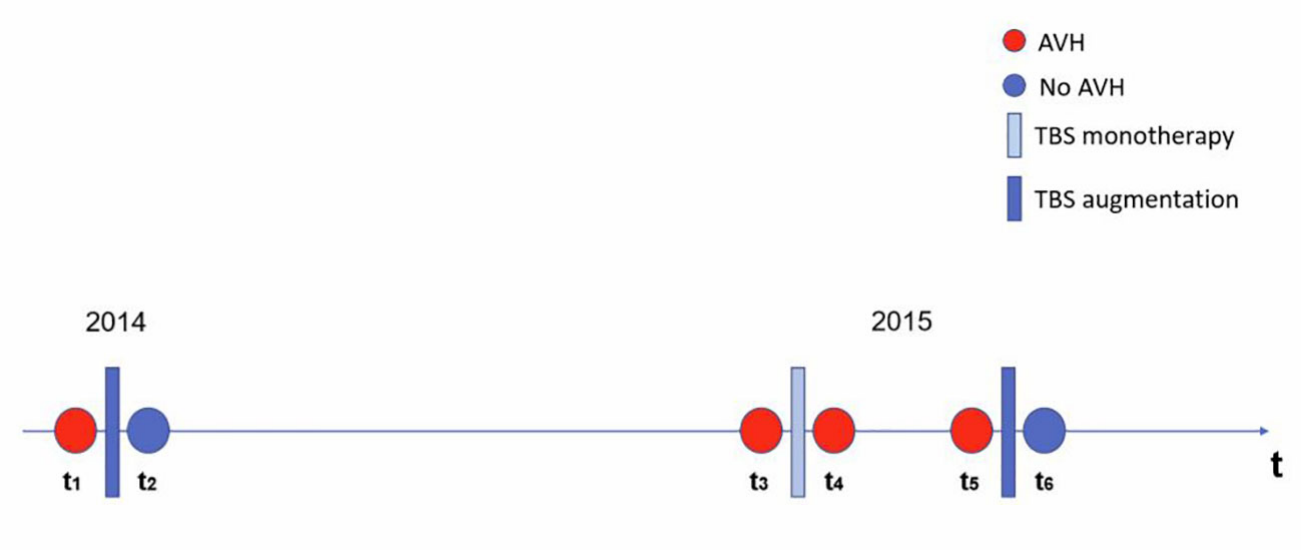
Schematic depiction of the functional magnetic resonance imaging (fMRI) data analysis design. The timeline (t) represents all MRI measurement times (t1–t6). TBS monotherapy is represented by a light blue bar, and TBS augmentation is represented by a dark blue bar. At t1, t3, t4, and t5 the patient experienced auditory-verbal hallucination (defined as “AVH”) indicated by a red colored circle. At t2, and t6, the patient experienced no symptoms of AVH (defined as “no AVH“) indicated by a blue colored circle.

Since, there are reports indicating brain activation changes of the auditory cortex after remission of AVHs in individuals with an early onset of SZ receiving rTMS (35), functional magnetic resonance imaging (fMRI) was performed to detect neural correlates of AVH (acute phase; the patient experience AVH) and no AVH (remission; the patient experienced no AVH). The MRI scans (structural and functional scans) were performed before and after each TBS course. In total, Mr. WF received six MRI scans. For analyses, functional data from the MRI scans prior the first, second, and third TBS series as well after the second TBS series (non-response to TBS monotherapy) were taken together (resulting in a total of 4 measurement times) and defined as “AVH” condition. Functional data from MRI scans after the first and the third TBS series were defined as “no AVH” condition (two MRI measurements). A schematically depiction is shown in **Figure 2**. During the second-level analysis, the conditions “AVH” and “no AVH” were compared at the whole-brain level employing z-statistics in consideration of multiple testing.

All imaging data were acquired on a 3-Tesla MR Scanner (Tim Trio, Siemens Medical Systems, Erlangen, Germany), equipped with a 12-channel head matrix receive Rx-coil, at the Department of Psychiatry and Psychotherapy, Philipps-University Marburg. Data acquisition consisted of a structural T1-weighted magnetization-prepared rapid gradient-echo sequence (MPRAGE) and a functional T2*-weighted single-shot echo-planar-imaging (SS-EPI) sequence sensitive to blood oxygenation level-dependent (BOLD) contrast with the following parameter settings:

- MPRAGE: repetition time (TR) = 1,900 ms, echo time (TE) = 2.26 ms, inversion time (TI) 900 ms, field of view (FoV) = 256 mm, 256 x 256 matrix, slice thickness (ST) = 1 mm, separation factor = 50 %, flip angle = 9°, 176 slices, parallel imaging (GRAPPA) with factor 2, bandwidth = 200 Hz/pixel.
- SS-EPI: 140 volumes, 37 slices, 5.3 mm effective slice thickness including a 6 % separation factor (i.e. interslice gap = 0.3 mm and slice thickness = 5 mm), repetition time (TR) = 1,580 ms, echo time (TE) = 30 ms, 64 x 64 matrix, field of view FoV = 192 x 192 mm^2^, bandwidth = 1,905 Hz/pixel, flip angle of α = 70°. Slices were recorded in ascending order, covering the whole brain, and were positioned transaxially parallel to the anterior–posterior commissural line (AC–PC).

Functional MR data processing was carried out using FEAT (FMRI Expert Analysis Tool) Version 6.0, part of FSL (FMRIB’s Software Library, www.fmrib.ox.ac.uk/fsl). The higher-level analysis was carried out using a fixed-effects model by forcing the random effects variance to zero in FLAME (FMRIB's Local Analysis of Mixed Effects) (36–38). Z (Gaussianised T/F) statistic images were thresholded using clusters determined by Z > 2.3 and a conservative cluster significance threshold of p = 0.05 (39).

During the functional MRI scan, an auditory stimulation paradigm similar to a previously described paradigm was used (40). The auditory stimulation was realized with tonal stimuli of alternating frequencies (ranging between 500 and 4,000 Hz) using an MRI-compatible headset. A total of 30 blocks were run. Each block lasted for 11 s with an inter-stimulus interval of 9 s. The auditory stimulation paradigm was used for all measurement times. The use of acoustic stimulation with alternating frequencies enables to stimulate the auditory cortex robustly (40). This was crucial because of the small case number of N = 1.

**Figure 3:**
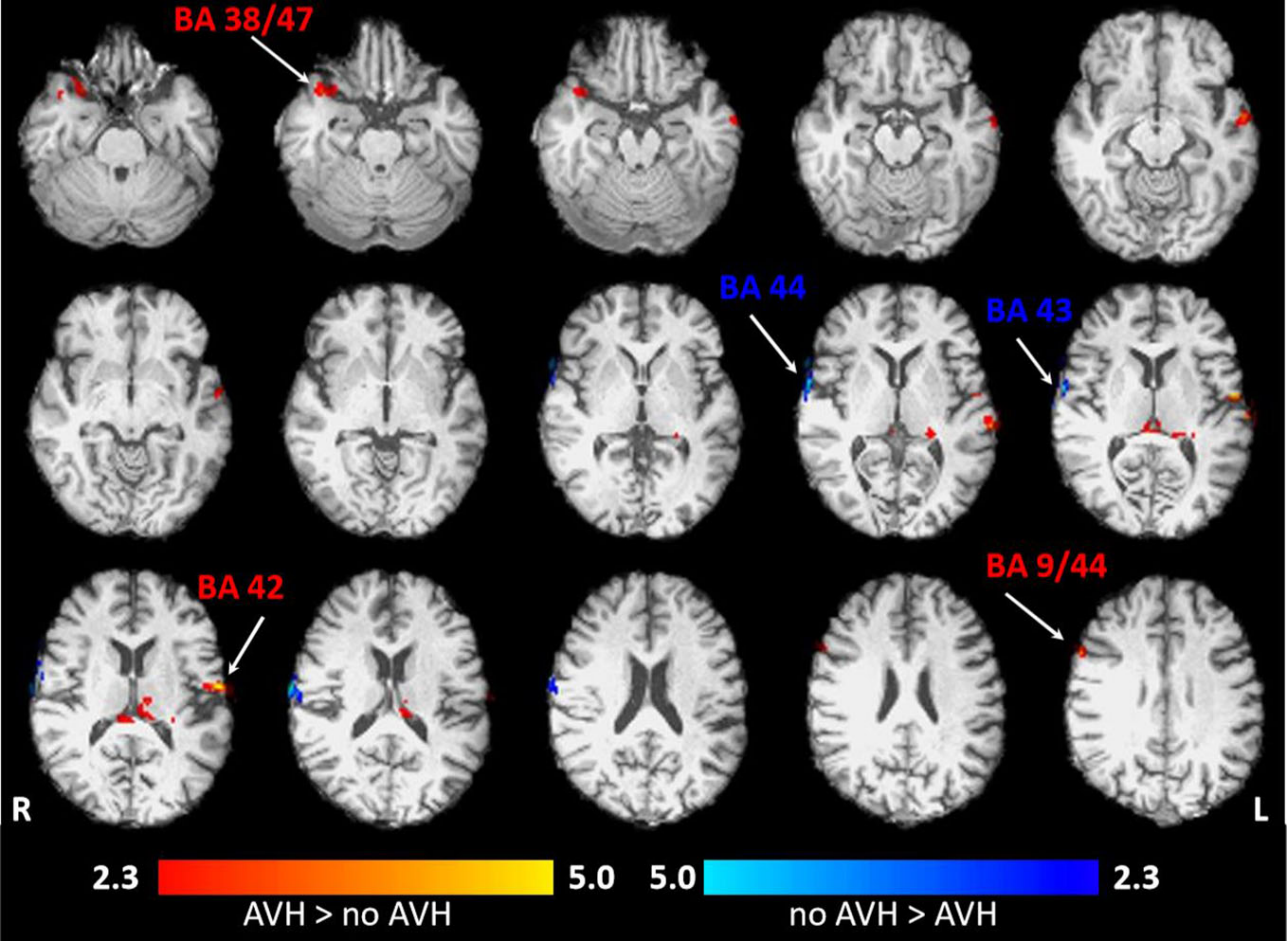
Localization and strength of brain activation during acoustic stimulation in Mr. WF. Axial view (superior view) is opposed to the observer's view: the left side of the brain corresponds to the right side. The red-colored clusters reflect the brain activation during auditory-verbal hallucination (AVH) > no AVH. The blue-colored clusters reflect brain activation. No AVH > AVH. *p *= 0.05, FWE (family-wise error)-corrected on cluster level. AVH, auditory verbal hallucination.

As can be seen in **Figure 3** and **Table 1**, the brain response during acoustic stimulation during the “AVH” as compared to the “no AVH” condition (AVH > no AVH) revealed stronger brain activation in several areas comprising the left acoustic primary cortex, the thalamus, and the right dorsolateral prefrontal cortex. At the time of “no AVH”, the brain activation was significantly reduced in the left acoustic primary cortex.

**Table 1:** Results of the analyses of BOLD responses for the contrasts (AVH > no AVH) and (no AVH > AVH).

		*Coordinates*				*Statistics*	
*Anatomical regions / BA*	H	x	y	*z*	*Cluster size*	*z-value*	*p-value*
***AVH > no AVH***							
Acoustic Primary Cortex / 42	L	−62	−10	12	179	4.98	<0.001
Thalamus / —	L	−8	−30	−10	171	3.06	<0.001
Pars Orbitalis / 47 Temporopolar / 38	L	30	20	−30	133	3.01	<0.001
Dorsolateral Prefrontal Cortex /9 Pars Opercularis/ 44	R	60	14	32	89	3.88	<0.05
***No AVH > AVH***							
Pars Opercularis / 44	R	64	16	0	169	7.16	<0.001
Primary Gustatory Cortex / 43	R	72	−8	14	112	5.89	<0.01

Results refer to gray matter. Coordinates of the peak voxels are listed in MNI (Montreal Neurological Institute) atlas space.

AVH, auditory-verbal hallucination; BA, Brodmann area; H, hemisphere; L, left; R, right.

P = 0.05, FWE (family-wise error)-corrected on cluster level.

## Discussion and Conclusions

To our knowledge, this is the first case reporting on the therapeutic effects of an add-on TBS treatment for auditory verbal hallucination in a patient with a VLOSLP. In the above-described case of the VLOSLP, pharmacological trials with atypical antipsychotics—considered to be the first-choice treatment in elderly patients (41)—led to a substantial side effect (i.e., impaired cognition, sedation, extrapyramidal-motor effects) or was insufficient. Partial remission of AVH could only be achieved using olanzapine, the best-tolerated substance for Mr. WF. Using the augmentation with TBS as applied over the left temporal lobe, we were able to achieve satisfactory improvement. After the relapse, we furthermore could replicate the observation of the olanzapine x TBS interaction.

### Possibly Common Pathway and Distant Origins

Our data might demonstrate that changes in brain activity pattern during tonal stimulation accompanied the improvement of AVH. The remission was associated with significantly reduced brain activation of the left auditory cortex while the brain activation in the right auditory cortex increased. This observation is in line with a previously published case study indication to a hyperactivation of the left auditory cortex during AVH in a 36-year-old woman (42). Also in line with our findings, further studies have shown, that rTMS/TBS treatment (when applied over the TP3 area) in SZ patients with AVH is related to brain activation change in the primary auditory cortex [e.g., (30)], a key brain region involved in tonal processing. In the case of Mr. WF, additionally to higher brain activation in the left primary auditory cortex (responsible for basal acoustic processing), the thalamus (filtering of sensory information) was also hyperactivated in the AVH condition, suggesting extended thalamocortical connectivity (43). Recent findings indicate that rTMS treatment when applied over the TP3—situated anatomically close to the temporoparietal junction—may impact the contribution of distant brain regions within the brain circuitry involved in language processing (44) without changing the functional coupling of them (45). This effect might provide the improvement of AVH symptoms in SZ patients (45). A similar mechanism may play a role in patients with VLOSLP.

Despite a high degree of similarity in terms of clinical symptomatology and treatment response between SZ and VLOSLP patients, the etiology of them was supposed to be different. While VLOSLP was assumed to have a neurovascular or/and neurodegenerative origin (46), SZ is considered to be an early-onset disease underlying neurodevelopmental aspects (47). The current case of VLOSLP might display similar neural alterations of auditory and language processing (associated with altered self-monitoring and misinterpretation of the own thoughts, which leads to AVH) as SZ patients do (48). Concerning VLOSLP, one can speculate that alterations within those neural circuits could be caused by vascular or neurodegenerative lesions or may possibly reflect stress-related accelerated brain aging (3)—an aspect which should be investigated in further research. The current case report also indicates that the treatment known to be efficient in SZ was also successful in VLOSLP. A positive effect of TBS on AVH relief in VLOSLP supplements earlier reports of TBS-induced improvement of AVH in younger patients (49, 50). This might be interpreted that the use of rTMS/TBS may effectively treat AVH for a wide range of age groups.

### Clinical Implications

Considering the world's growing and increasingly aging population, VLOSLP might become more important in future clinical settings (51). Up to two-thirds of VLOSLP patients respond to antipsychotic medication (52, 53), but with increasing age, the risk of adverse effects of medication increases significantly (54). Elderly patients show increased sensitivity to pharmacological interventions and are more frequently affected by severe adverse responses to antipsychotics (cognitive decline, sedation, extrapyramidal motor effects), resulting from altered metabolic functions, co-morbidities, and medical interactions (53, 55). In the case of Mr. WF, the cerebral arteriosclerotic encephalopathy, and the possible neurodegenerative disease were accompanied by hypersensitivity of cognitive and extrapyramidal side effects. Antipsychotic treatment in the elderly population should therefore often be managed as a smaller daily dose that may reduce their efficacy. In comorbid dementia, antipsychotic medication was also reported to be associated with inferior efficacy and potential risks, including a shorter life expectancy (56, 57). Thus, more therapeutic options are needed. As recently demonstrated for AVH treatment in younger SZ patients (22) and in elderly depressed patients (58), non-invasive brain stimulation techniques (e.g., rTMS and TBS) are beneficial in terms of the tolerance, side-effect profile, and patient's acceptance. Therefore, they can be integrated into everyday clinical practice and might become an appropriate option to treat affective and psychotic symptoms occurring, particularly in elderly patients. The benefits of the well-tolerated rTMS/TBS as an add-on treatment option may allow the use of a lower antipsychotic dosage for treating AVH and will result in a lower burden of adverse side effects on the patients' health, especially those of older age.

### Limitations

This case study comprises several strengths and limitations. The transferability of therapeutic effects for VLOSLP patients is strongly limited by the naturalistic single-case design of the present study, although favorable treatment effects were found in the current case. There is often a high variance of fMRI findings even though robust activation of the auditory cortex could be found after tonal stimulation. However, MRI findings are currently rare in investigating VLOSLP. Therefore, we encourage other groups to report their observation providing a better understanding of pathophysiology underlining VLOSLP in the future.

## Conclusion

The clinical case described in the current manuscript indicates that the novel non-invasive brain stimulation techniques (e.g., TBS) can be used as a tool facilitating medicament effects, particularly for managing AVHs in VLOSLP. Considering the responsiveness to the same treatment (antipsychotic medication and rTMS/ TBS), and our imaging data possibly indicating to disturbed auditory processing in VLOSLP, we speculate that a common pathophysiological pathway may be responsible for auditory verbal hallucinations in SZ and VLOSLP. Given methodical limitations of the current case study, this assumption should be treated with caution and be addressed in future research. Combining conventional (i.e., antipsychotic medication) and novel (brain stimulation) therapy approaches may offer an improvement treatment by greater effectiveness and fewer side effects. Further case series and prospective controlled studies on larger collectives are required to validate this outcome.

## Data Availability Statement

The datasets used and analyzed during the current study are available from the corresponding author on reasonable request.

## Ethics Statement

The patient and the patient's son gave his consent for publication of the study.

## Author Contributions

MZ, UD, TK, A-FH, and JS were responsible for data acquisition. JS and RZ were responsible for data analyses. RZ and MZ drafted the manuscript, including all tables and figures. RZ, MZ, JS, TK, UD, and A-FH contributed to the interpretation of the data and critically revised the manuscript. All authors contributed to and have approved the final manuscript.

## Funding

The work was supported by the German Research Foundation (grant number DFG FOR2107 DA1151/5-1, DA1151/5-2, SFB-TRR58, C09, and Z02), the Interdisciplinary Centre for Clinical Research of the University of Muenster, Germany (grant number IZKF FG4 and Dan3/012/17), the University Medical Center Giessen and Marburg (grant number UKGM, 27/2015 MR), the Rhoen-Klinikum AG (grant number RKA, FI22/2015), and the von Behring-Roentgen-Foundation (grant number BRS, 64-0016). The funding organizations had no influence on the design and conduct of the study, collection, management, analysis, and interpretation of the data, preparation, review, or approval of the manuscript.

## Conflict of Interest

In 2015, MZ received financial support for an educational program from Lundbeck, Servier, Actelion, MagVenture, Mag and More, Localite, Inomed, Sooma Oy, Brainsway/Tolko, and NeuroConn. However, it did not influence the content of this manuscript.

The remaining authors declare that the research was conducted in the absence of any commercial or financial relationships that could be construed as a potential conflict of interest.

## Abbreviations

AVH, auditory-verbal hallucination; rTMS, repetitive transcranial magnetic stimulation; SGA, second-generation antipsychotics; SZ, schizophrenia; TBS, theta-burst stimulation; VLOSLP, very-late onset schizophrenia-like psychosis.
