# Supplementary material for: Theta-Burst Stimulation for Auditory-Verbal Hallucination in Very-Late-Onset Schizophrenia-Like Psychosis—A Functional Magnetic Resonance Imaging Case Study
Source: Front Psychiatry. 2020 Apr 20;11:294. doi: 10.3389/fpsyt.2020.00294 (PMC7212466; doi:10.3389/fpsyt.2020.00294)
Supplement: Supplementary file 1 [file DataSheet_1.pdf]

Theta-burst stimulation (TBS) for auditory-verbal hallucination in very-late-onset schizophrenia-like psychosis – An fMRI case study.

Supplementary data: detailed description of the case.

Rebecca Zöllner, Anne-Friederike Hübener, Udo Dannlowski, Tilo Kircher, Jens Sommer, Maxim Zavorotnyy

The first psychotic episode in 2006

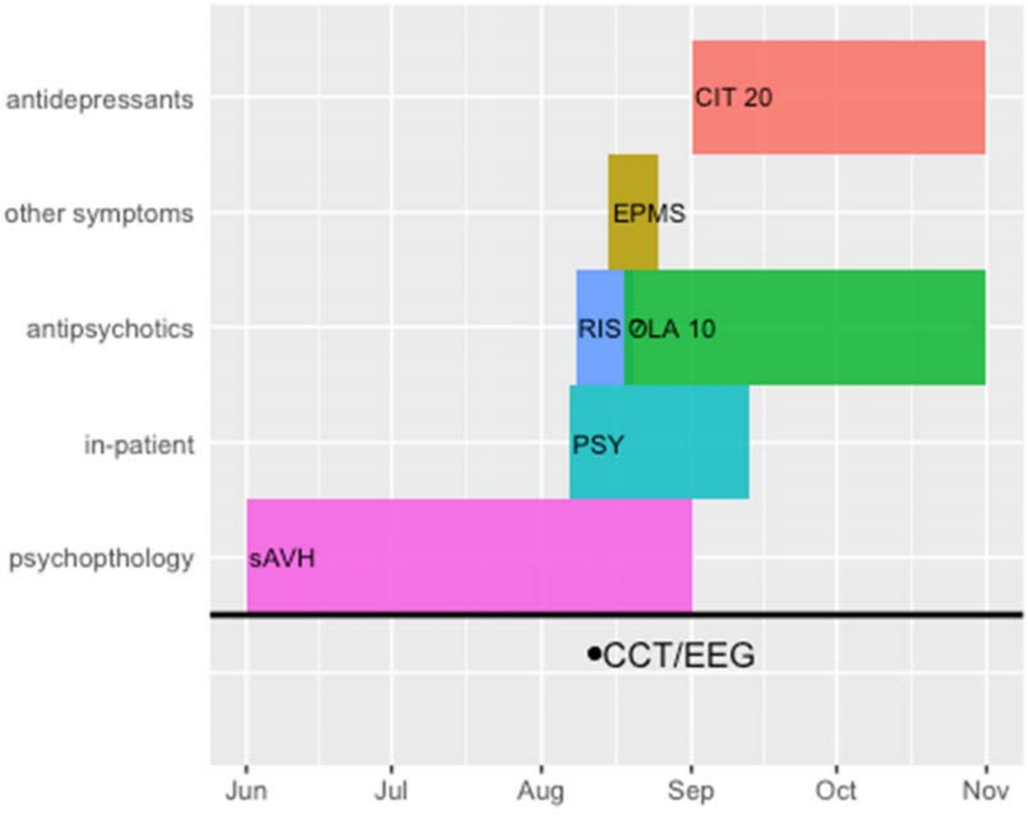

Symptoms:

- AVH – Auditory verbal hallucinations (approximately reconstructed from the medical report).
- sAVH – Severe AVH, somatosensory hallucinations, paranoid delusions.
- EPMS – Extra-pyramidal-motoric symptoms.

In-patient treatment:

- PSY – In-patient in the Depatment of Psychiatry of the Eichhof Hospital Lauterbach, Germany.

Diagnostics:

- CCT/EEG – Cranial computer tomography and electroencephalography: both normal.

Medication:

- RIS ? – Antipsychotic medication with risperidone, an unknown daily dosage.
- OLA 10 – Antipsychotic medication with olanzapine, 10 mg daily.
- CIT 20 – Antidepressant medication with citalopram, 20 mg daily.

The second psychotic episode in 2014

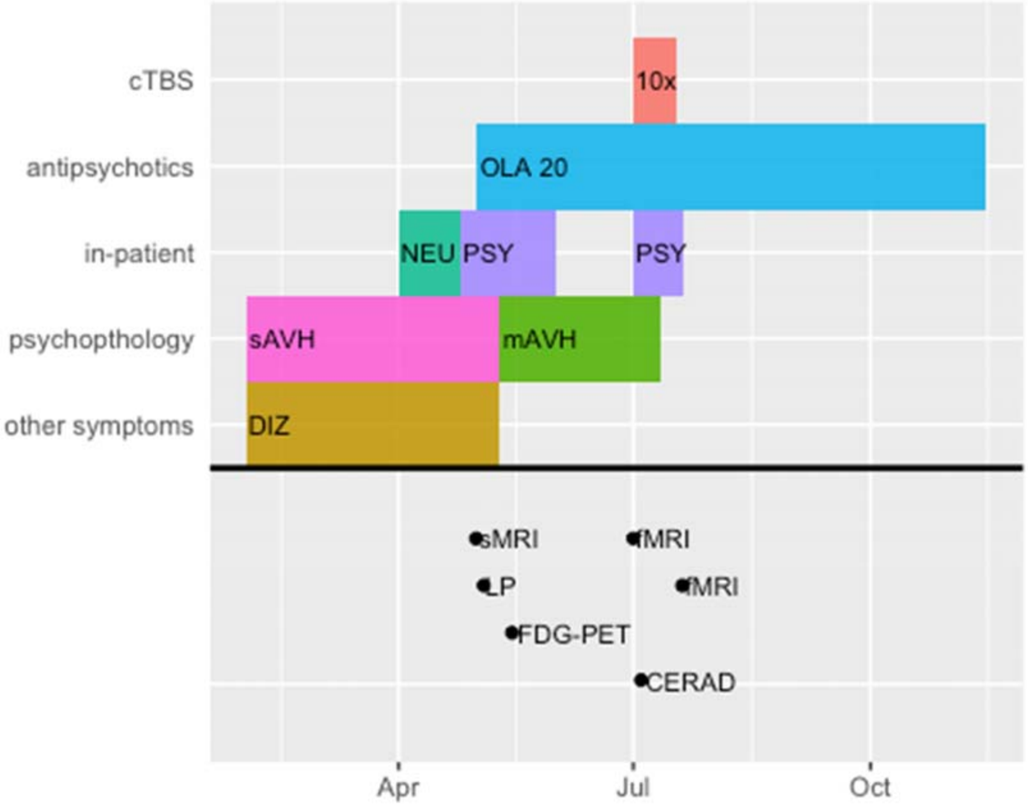

Symptoms:

- AVH – Auditory verbal hallucinations (examined using targeted examination of AVH, see Box S1).
- sAVH – Severe AVH, somatosensory hallucinations, paranoid delusions.
- mAVH – moderate AVH: subjective reduced frequency and intensity of AVH.
- DIZ – Dizziness.

Box S1. Targeted examination of AVH

**Questions**
Do you hear voices?
If so, please describe them. Are the voices associated with other sensory perceptions? How much do you feel disturbed by the voices? Moderate or substantial?
If not, when was the last time you heard the voices?

In-patient treatment:

- PSY – In-patient in the Department of Psychiatry and Psychotherapy of the Philipps-University Marburg, Germany.
- NEU – In-patient in the Department of Neurology of the Philipps-University Marburg, Germany.

Diagnostics:

- sMRI – Structural magnetic resonance imaging revealed a subcortical atherosclerotic encephalopathy.
- LP – Lumbal punction. Analysis of the cerebrospinal fluid revealed no pathologic signs, ß-amyloid, and tau-proteins were normal.
- FDG-PET – Positron-emission tomography using F-18-FDG was suspicious for Alzheimers' disease.
- fMRI – Functional MRI using the tonal stimulation paradigm.
- CERAD – CERAD test battery revealed a mild neurocognitive decline (see Table S1).

Table S1. CERAD Testing Battery, July 3rd 2014

Variables	Min.	Max.	Raw Value	z-Value
Verbal Fluency, animal naming	0	-	5	-3.14
Boston Naming Test, 15 items	0	15	9	-2.93
Mini-Mental State Examination	0	30	23	-3.18
Word List Learning Total	0	30	5	-4.17
Word List Learning 1st trial	0	10	1	-2.55
Word List Learning 2nd trial	0	10	2	-3.25
Word List Learning 3rd trial	0	10	2	-4.36
Word List Delayed Recall	0	10	0	-3.07
Word List Intrusions	0	-	0	0.83
Word List Savings	0	-		
Word List Recognition	0	100%	70%	-2.95
Constructional Praxis	0	11	8	-2.07
Constructional Praxis Recall	0	11	4	-2.17
Constructional Praxis Savings	0	100%	50%	-1.60
Trail Making Test A	0	180	90	-1.86
Trail Making Test B	0	300	-	-
Trail Making Test A/B	0	-	-	-
Phonemic Fluency, S-words	0	-	-	-

The z-values have been corrected concerning age, gender, and years of education (sample CERAD: N = 1100; sample phonematic fluency and trail making test A + B: N = 604). Ranges: age CERAD 49 - 92; age phonemic fluency and trail making test: 50-88; years of education (all tests): 7-20 years.

Medication:

- OLA 20 – antipsychotic medication with olanzapine, start with 2.5 mg, at the discharge 20 mg daily

Repetitive transcranial magnetic stimulation:

- cTBS – continues theta-burst stimulation over PT3 according to the 10-20-system.
- 10x – 10 days cTBS.

Before the third psychotic episode in 2015

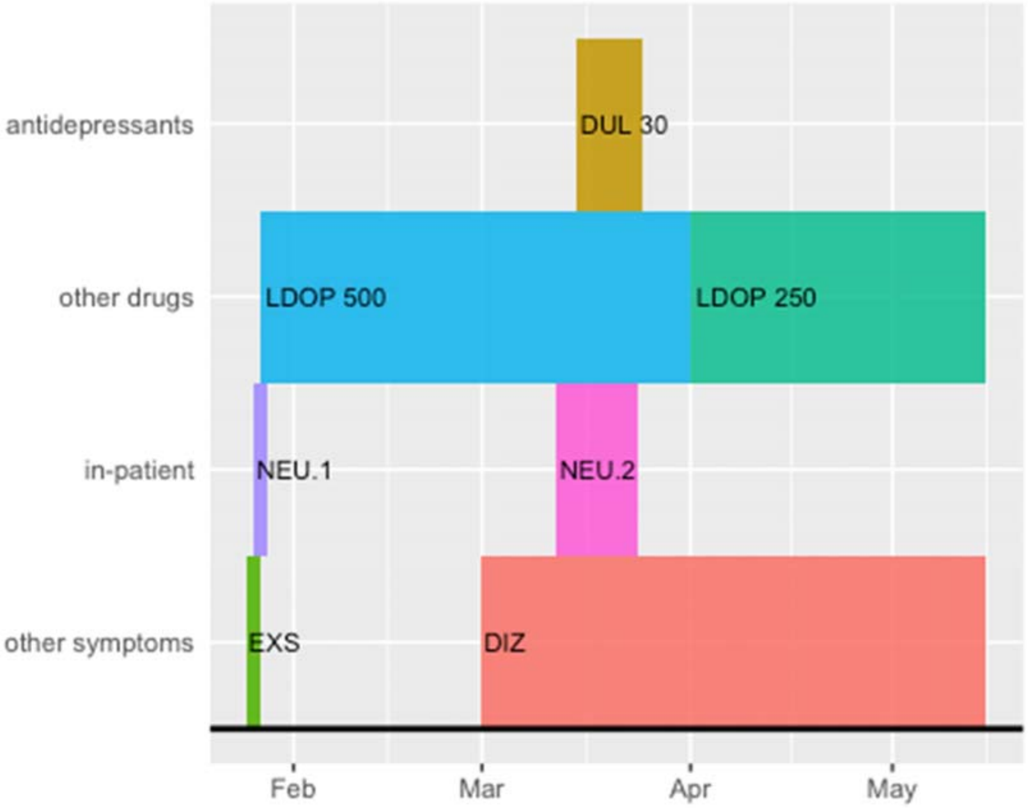

Symptoms:

- EXS – Exsiccosis.
- DIZ – Dizziness.

In-patient treatment:

- NEU.1 – In-patient in the Department of Neurology of the Alsfeld Hospital, Germany.
- NEU.2 – In-patient in the Department of Neurology of the Hephata Hospital in Schwalmstadt, Germany.

Medication:

- LDOP 500 – Medication with l-DOPA, 500 mg daily.
- LDOP 250 – Medication with l-DOPA, 250 mg daily.
- DUL 30 – Antidepressant medication with duloxetine, 30 mg daily.

Third psychotic episode in 2015

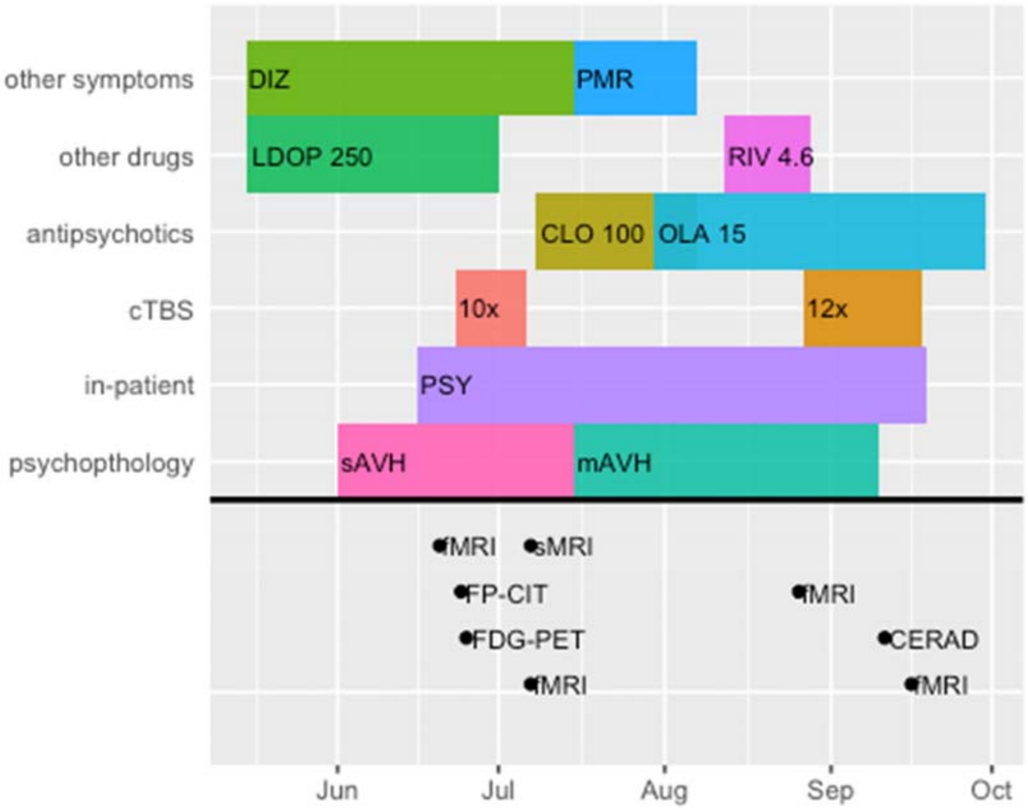

Symptoms:

- AVH – Auditory verbal hallucinations (examined using targeted examination of AVH, see Box S1).
- sAVH – Severe AVH, somatosensory hallucinations, paranoid delusions.
- mAVH – Moderate AVH: reduced frequency and intensity of AVH.
- DIZ – Dizziness.
- PMR – psycho-motor retardation.

In-patient treatment:

- PSY – In-patient in the Department of Psychiatry and Psychotherapy of the Philipps-University Marburg, Germany.

Diagnostics:

- fMRI – Functional MRI using the tonal stimulation paradigm.
- FP-CIT – DAT-Scan was unsuspicious for idiopathic Parkinsons' syndrome.
- FDG-PET – Positron-emission tomography using F-18-FDG was suspicious for Alzheimers' disease with a tendency of progress.
- sMRI – Structural magnetic resonance imaging revealed a subcortical atherosclerotic encephalopathy and signs of global brain atrophy.

- CERAD – CERAD test battery revealed mild neurocognitive decline (see Table S2) with a tendency of progress if compared to July 2014.

Table S2. CERAD Testing Battery, September 11th 2015

Variables	Min.	Max.	Raw Value	z-Value
Verbal Fluency, animal naming	0	-	5	-2.87
Boston Naming Test, 15 items	0	15	7	-3.49
Mini-Mental State Examination	0	30	18	-4.84
Word List Learning Total	0	30	7	-3.28
Word List Learning 1st trial	0	10	2	-1.60
Word List Learning 2nd trial	0	10	2	-3.05
Word List Learning 3rd trial	0	10	3	-3.36
Word List Delayed Recall	0	10	2	-1.55
Word List Intrusions	0	-	1	-0.63
Word List Savings	0	-	67%	-0.62
Word List Recognition	0	100%	56%	-3.78
Constructional Praxis	0	11	7	-2.42
Constructional Praxis Recall	0	11	5	-1.54
Constructional Praxis Savings	0	100%	71%	-0.61
Trail Making Test A	0	180	121	-2.38
Trail Making Test B	0	300	300	-1.51
Trail Making Test A/B	0	-	2.5	0.57
Phonemic Fluency, S-words	0	-	0	-3.10

The z-values have been corrected concerning age, gender, and years of education (sample CERAD: N = 1100; sample phonematic fluency and trail making test A + B: N = 604). Ranges: age CERAD 49 - 92; age phonemic fluency and trail making test: 50-88; years of education (all tests): 7-20 years.

Medication:

- LDOP 250 – Medication with l-DOPA, 250 mg daily.
- CLO 100 – Antipsychotic medication with clozapine, start with 12.5 mg, maximal dosage 100 mg daily, tapered out due to a psychomotor retardation.
- OLA 15 – Antipsychotic medication with olanzapine, start with 2.5 mg, at the discharge 15 mg daily.
- RIV 4.6 – Treatment trial with rivastigmine, 4.6 mg daily.

Repetitive transcranial magnetic stimulation:

- cTBS – Continues theta-burst stimulation over PT3 according to the 10-20-system.
- 10x – 10 days with iTBS (monotherapy).
- 12x – 12 days with iTBS (add-on).
